# Spatial–temporal combination and multi-head flow-attention network for traffic flow prediction

**DOI:** 10.1038/s41598-024-60337-7

**Published:** 2024-04-26

**Authors:** Lianfei Yu, Wenbo Liu, Dong Wu, Dongmei Xie, Chuang Cai, Zhijian Qu, Panjing Li

**Affiliations:** 1 School of Computer Science and Technology, Shandong University of Technology, Zibo, 255000 China; 2 Inspur (Jinan) Data Technology Co., Ltd, Jinan, 250000 China

**Keywords:** Computer science, Information technology

## Abstract

Traffic flow prediction based on spatial–temporal data plays a vital role in traffic management. However, it still faces serious challenges due to the complex spatial–temporal correlation in nonlinear spatial–temporal data. Some previous methods have limited ability to capture spatial–temporal correlation, and ignore the quadratic complexity problem in the traditional attention mechanism. To this end, we propose a novel spatial–temporal combination and multi-head flow-attention network (STCMFA) to model the spatial–temporal correlation in road networks. Firstly, we design a temporal sequence multi-head flow attention (TS-MFA), in which the unique source competition mechanism and sink allocation mechanism make the model avoid attention degradation without being affected by inductive biases. Secondly, we use GRU instead of the linear layer in traditional attention to map the input sequence, which further enhances the temporal modeling ability of the model. Finally, we combine the GCN with the TS-MFA module to capture the spatial–temporal correlation, and introduce residual mechanism and feature aggregation strategy to further improve the performance of STCMFA. Extensive experiments on four real-world traffic datasets show that our model has excellent performance and is always significantly better than other baselines.

## Introduction

Intelligent Transportation System (ITS)^1^ plays an important role in road traffic management. It combines some advanced science and technology such as information technology and sensor technology^2^, and is effectively applied to traffic management and transportation. Traffic prediction is one of the important components of ITS, and it is also the issue that many researchers are scrambling to study. With the continuous progress and development of the times, road transportation plays an increasingly important role in people’s lives, and the pressure on road traffic is also increasing. On the other hand, traffic accidents and road congestion are also happened more frequently, which greatly affects people’s travel efficiency and safety. Especially on highways with high speed, road congestion will seriously affect road traffic and even cause traffic accidents. With the maturity of sensor technology, the collection and storage of traffic data are more convenient. Traffic flow, traffic speed, and traffic occupancy data can be collected and used in traffic prediction research. Among them, traffic flow is the most intuitive indicator to reflect road conditions.

**Figure 1:**
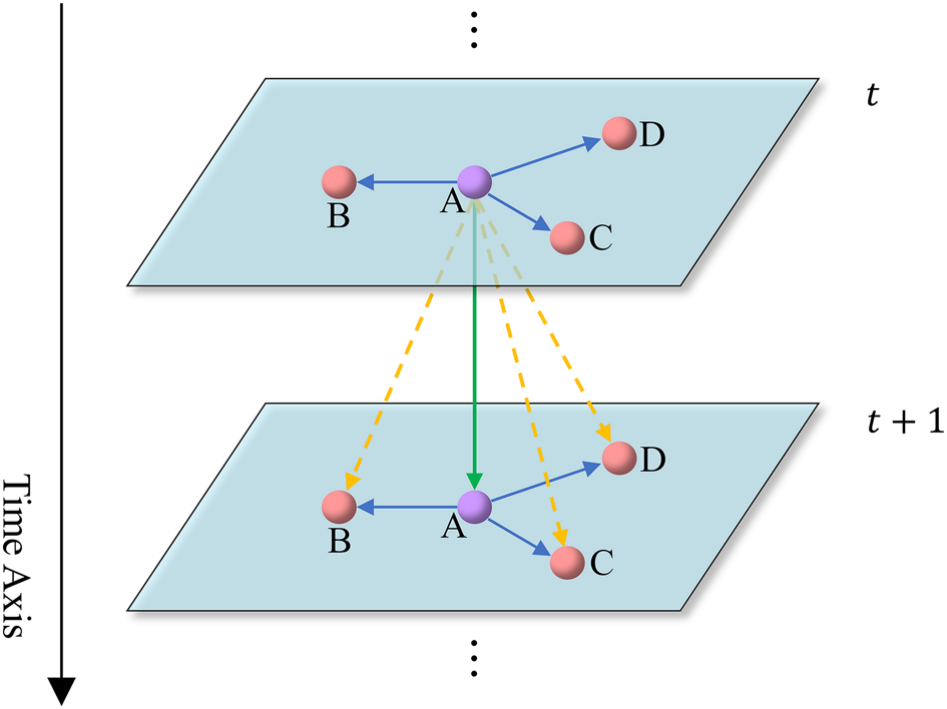
Spatial–temporal correlation.

Traffic flow prediction is a typical spatial–temporal data prediction problem^3–5^. How to capture the spatial–temporal correlation from traffic data is a major challenge for traffic flow prediction. Temporal correlation is that different traffic conditions will occur at different times. For example, the traffic flow on the road in the morning, noon, and evening is usually larger than other time periods. In holidays, the traffic flow is also different from that in normal working days. If a traffic accident happens at a certain time and causes traffic congestion, then the traffic flow of this section in the next time period will be affected. Spatial dependence means that if different roads are connected to each other, the traffic state of a certain road will have a range of effects on the connected roads. Specifically, the upstream road will affect the downstream road, and the downstream road will in turn affect the upstream road because of the feedback effect^6^. As shown in Fig. 1: The purple circle represents node A, and the red circles B, C, and D represent nodes directly connected to node A in space. The blue arrow indicates spatial dependence, the green arrow indicates temporal correlation, and the yellow arrow indicates spatial–temporal correlation. $t$ and $t+1$ are two adjacent moments on the time axis. Specifically, node A will affect the nodes B, C, and D connected to it in space at moment $t$, and will also affect the node A itself at moment $t+1$. Due to the spatial–temporal correlation, the node A at moment $t$ will even affect the nodes B, C, and D at moment $t+1$.

In recent years, deep learning have been widely applied to traffic flow prediction, such as Recurrent Neural Network (RNN) and its variant Long Short-Term Memory network (LSTM)^7^. However, these methods have a slower calculation speed, and there is a risk of gradient disappearance or gradient explosion when capturing the correlation of long-term sequences. Moreover, they can only capture the temporal correlation of sequences and cannot process the spatial information of traffic data, which leads to their poor prediction performance. Researchers also try to capture the spatial dependence of traffic data by using the spatial network topology generated by the actual spatial connection state of the road sensor. Graph Convolutional Network (GCN) is the most commonly used method for processing spatial information. The spatial–temporal synchronous graph convolutional network (STSGCN)^8^ was proposed to capture the complex spatial–temporal heterogeneities in the sequence by generating multiple local spatial–temporal subgraphs, but it only considered the local correlation of spatial–temporal information and ignored the global spatial–temporal correlation. The spatial–temporal graph ODE networks (STGODE)^9^ uses ordinary differential equation based on tensor to capture the spatial–temporal correlation of information, but its ability to capture spatial–temporal correlation can still be improved. The spatial–temporal dynamic semantic graph neural network (STDSGNN)^10^ captures spatial–temporal correlation by combining multi-head graph attention and full convolution, but it does not consider the influence of inductive biases and quadratic complexity in attention. The spatio-temporal shared GRU (STSGRU)^11^ improves the prediction scale by designing a shared weight mechanism, and captures the long-term dependence on the time dimension by modeling periodicity, but the type of captured long-term dependence is relatively single.

Attention mechanism is often applied to the related research of traffic flow prediction^12–14^. It can adaptively calculate the weights of different positions according to the characteristics of different positions in the input sequence, thereby concentrating more attention on the relatively important positions. Although the attention mechanism can improve the prediction performance of the model by capturing the key information in the sequence, it usually introduces specific inductive biases in order to avoid attention degradation, which reduces the versatility of the model to a certain extent. In addition, the calculation burden corresponding to the attention weight will increase quadratically when processing long sequence data, causing the quadratic complexity problem^15^.

Aiming at the above limitations, we proposed the spatial–temporal combination and multi-head flow-attention network (STCMFA). STCMFA introduces the idea of flow conservation into the attention mechanism, so that the model can form a natural competition mechanism without specific inductive biases. The unique competition mechanism in STCMFA can effectively solve the quadratic complexity problem caused by calculating the attention weight in the traditional attention mechanism. In addition, it combines the temporal sequence model with the multi-head flow attention, which can capture a variety of changes and long-term dependencies in the time dimension, thus improving the prediction performance of the model. Our contributions are summarized as follows:

1. We design a temporal sequence multi-head flow attention (TS-MFA), in which the unique source competition mechanism and sink allocation mechanism replace the traditional attention weight calculation module, thus avoiding the impact of specific inductive biases.
2. We use GRU to replace the linear transformation in traditional attention, thereby enhancing the ability of attention to capture temporal correlation when dealing with long-term sequences.
3. We combine TS-MFA with GCN to capture complex spatial–temporal correlations. The residual mechanism and feature aggregation strategy are introduced into STCMFA to further improve its performance.
4. Extensive experiments are conducted on four real-world traffic datasets. The experimental results show that our model always outperforms the baseline models.

The structure of this paper is as follows: “Related work” section introduces the related work of traffic flow prediction in recent years. “Methodology” section gives the relevant definitions and specific method implementations. A large number of experiments were carried out in the “Experiments” section, and the experimental results were analyzed. Finally, “Conclusions” section summarizes this work.

## Related work

### Traffic flow prediction

Traffic flow prediction is to use the spatial–temporal data collected by road sensors to make as accurate a prediction as possible for the traffic state in the future. There are many methods have been presented for traffic flow prediction. The early prediction methods include statistical models such as Historical Average model (HA)^16^ and Autoregressive Integrated Moving Average (ARIMA)^17^. The statistical models are mainly based on statistical knowledge for mathematical derivation. Although it does not require a lot of training and iteration, it is not suitable for traffic flow prediction of complex road sections with a large amount of data. The related methods of machine learning were also applied to traffic flow prediction, such as K-Nearest Neighbor algorithm (KNN)^18^. This method needs to find the nearest neighbour value, so the calculation speed is slow, and the selection of K value will also affect the prediction accuracy. The Support Vector Machine algorithm (SVM)^19^ is difficult to find the optimal parameters when the amount of data is large. Later, researchers began to try to use deep learning methods for traffic flow prediction, including RNN. In order to solve the problems of gradient disappearance and gradient explosion, variant LSTM and gated recurrent unit (GRU)^20^ based on RNN were proposed. However, these models can only capture temporal correlation and cannot process spatial information. The fully-connected LSTM (FC-LSTM)^21^ integrates full connectivity on the basis of LSTM to model spatial–temporal correlation. Convolutional Long Short-Term Memory network (ConvLSTM)^22^ employs Convolutional Neural Network (CNN) to replace the Hadamard product operation in LSTM, and the prediction effect is better than FC-LSTM. Zhang et al.^23^ proposed the deep spatio-temporal residual networks (ST-ResNet), which combines with deep residual network to predict crowd flow. However, these above methods were only applicable to grid data, and cannot deal with complex spatial topology information. Yao et al.^24^ proposed the deep multi-view spatial–temporal network (DMVST-Net), which uses local CNN to capture the local features of the region and expresses the semantic information of the region features by constructing a region graph. Xu et al.^25^ proposed the spatiotemporal convolution network based on long-term, short-term, and spatial features (GCGRU), which uses GNN and RNN to capture the spatial dependence and temporal correlation, respectively.

### Graph convolutional network

Traditional CNNcannot to process graph-structured data well. GCN based on CNN can process non-Euclidean graph-structured data. In recent years, many researchers have tried to use GCN for traffic flow prediction. For example, the heuristic linear method proposed by Niepert et al.^26^ to select the neighborhood of each node has achieved good prediction results. The diffusion convolutional recurrent neural network (DCRNN) proposed by Li et al.^27^ models traffic flow in the form of diffusion, capturing the spatial dependence of directed graphs. Yu et al.^28^ proposed spatio-temporal graph convolutional networks (STGCN), which uses graph convolution to capture spatial dependence and uses one-dimensional convolution to capture temporal correlation. The model is constructed with pure convolution structure, and the small number of parameters leads to its very fast training speed. Wu et al.^29^ proposed a new graph convolutional network architecture (Graph WaveNet) for spatial–temporal graph modeling. By developing a new adaptive dependency matrix to accurately capture the hidden spatial dependencies in traffic data, and using stacked 1D-CNN components to expand the receptive field. Bai et al.^30^ proposed an adaptive graph convolutional recurrent network (AGCRN), in which two adaptive modules are designed: the node adaptive parameter learning module is used to capture the specific traffic patterns of different road nodes, and the adaptive graph generation module is used to automatically capture dynamic spatial dependencies. Song et al.^8^ proposed a spatial–temporal synchronous graph convolutional networks (STSGCN), which constructs multiple local spatial–temporal subgraphs and captures local spatial–temporal correlation through spatial–temporal synchronization modeling mechanism, but it ignores the complex spatial–temporal correlation in the global. Fang et al.^9^ proposed spatial–temporal graph ODE networks (STGODE), which uses tensor-based ordinary differential equations to capture complex spatial–temporal correlation. Lan et al.^31^ proposed a novel dynamic spatial–temporal aware graph neural network (DSTAGNN), which replaces the traditional predefined graph by constructing a dynamic spatial–temporal perception graph based on data-driven strategy, and obtains a wide range of dynamic temporal correlation from multiple receptive field features through multi-scale gated convolution. Tan et al.^32^ proposed a spatial–temporal graph product convolutional network (STGPCN), which can obtain different cross-spacetime graphs by defining different forms of graph product, so as to capture complex cross-spacetime node relationships.

### Attention mechanism

In recent years, attention mechanism has been widely used in speech recognition and traffic flow prediction^33,34^. The attention mechanism is to filter out the information that is critical to the current task by assigning different weights to the input data, and then use the limited resources to focus on critical information. The attention mechanism can not only improve the computational speed, but also model the variable long sequence, which makes it have ability to capture long-term dependence. Xu et al.^35^ proposed an image caption generator based on two attention mechanisms: “soft” certainty and “hard” randomness, and analyzed the prediction results in a visual way. The graph attention network proposed by Velickovic et al.^36^ combines graph convolution with self-attention to process graph structure data and achieves good prediction results. Guo et al.^37^ proposed attention based spatial–temporal graph convolutional networks (ASTGCN), which proposes a novel spatial–temporal attention mechanism to adaptively capture dynamic spatial–temporal correlation. However, a single attention mechanism can only map features into one space, which leads to its weak modeling ability. Li et al.^38^ proposed a transformer-enhanced spatial temporal graph neural network (DetecorNet), which models the temporal correlation of long-distance and short-distance by constructing multi-view temporal attention module and dynamic attention module. Zhang et al.^39^ proposed a self-attention based ChebNet recurrent network (SACRN), which captures spatial–temporal correlation by designing a spatial self-attention mechanism and fusing it with Chebyshev network and LSTM. Qin et al.^40^ proposed the linear transformer based on reweighting mechanism, which achieved excellent performance, but reduced the versatility of the model. Therefore, Wu et al.^41^ introduced flow conservation into the attention mechanism and proposed the flow attention mechanism, which improved the versatility of the model and reduced the complexity.

Although the above attention variants improved by traditional attention mechanisms solve a variety of problems, they still lack the ability to capture spatial–temporal correlation. Inspired by these studies, we consider combining flow attention mechanism, temporal model, and graph convolutional network to capture more comprehensive spatial–temporal correlation. At the same time, the multi-head attention mode is retained to improve the expression ability of the model.

## Methodology

### Preliminaries

#### Traffic network

**Figure 2:**
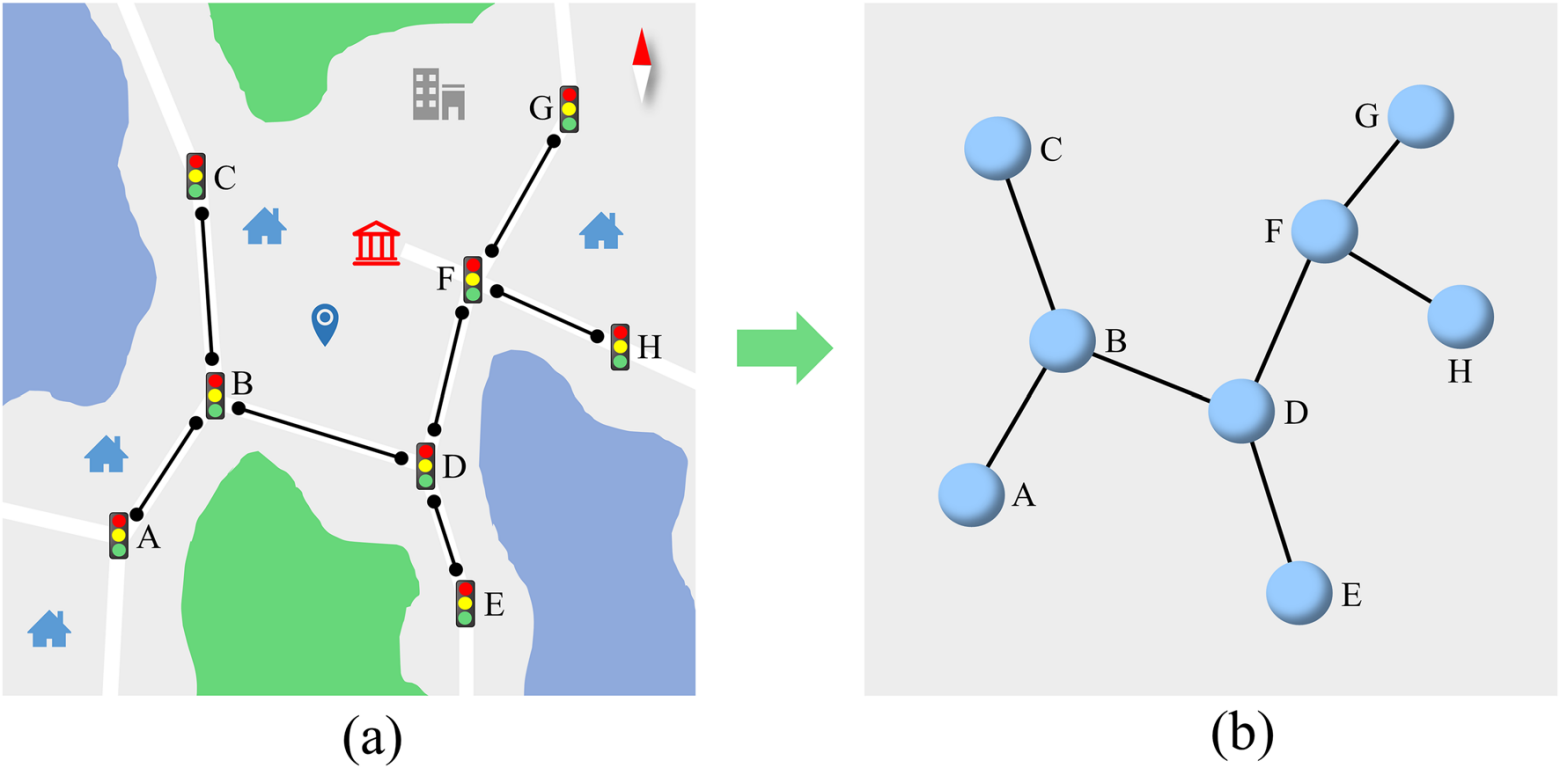
Road network generates spatial topography.

As shown in Fig. 2, the corresponding road network topology is generated according to the road connection relationship between each sensor in the actual road network, which is represented by $G=\left(V,E,A\right)$, where $V=\left\{{v}_{1},{v}_{2},\dots ,{v}_{N}\right\}$ represents the set of all nodes (road sensors) in the entire road network, and $N$ represents the number of nodes. $E$ is a set of edges representing the connectivity between nodes. If there are two nodes connected by a road, then there is an edge in $G$ connecting the two nodes. $A\in {R}^{N\times N}$ is the adjacency matrix, ${a}_{ij}$ is an element in $A$, which represents the spatial connection state between node ${v}_{i}$ and node ${v}_{j}$. If there exist ${v}_{i},{v}_{j}\in V$ and $\left({v}_{i},{v}_{j}\right)\in E$, then the value of ${a}_{ij}$ is 1, otherwise it is zero.

### Construction of time characteristics

The time characteristics of all nodes in the topological graph $G$ are represented by $X\in {R}^{N\times L\times F}$, where $L$ denotes the total length of time series of each node, $F$ denotes the number of types of characteristics of nodes. The process of traffic flow prediction can be described by Eq. (1):

1 $$\begin{array}{c}\left({X}_{t+1},{X}_{t+2},\dots ,{X}_{t+{T}{\prime}}\right)=F\left[\left({X}_{t-T+1},{X}_{t-T+2},\dots ,{X}_{t}\right);G\right],\end{array}$$

where $t$ denotes a moment, $T$ is the length of historical traffic data, and $T{\prime}$ is the length of future traffic data to be predicted. Given continuous time steps $\left({X}_{t-T+1},{X}_{t-T+2},\dots ,{X}_{t}\right)$ and topological graph $G$, by training a model $\mathcal{F}$, we can predict the future $T{\prime}$ continuous time steps $\left({X}_{t+1},{X}_{t+2},\dots ,{X}_{t+{T}{\prime}}\right)$.

### STCMFA model

**Figure 3:**
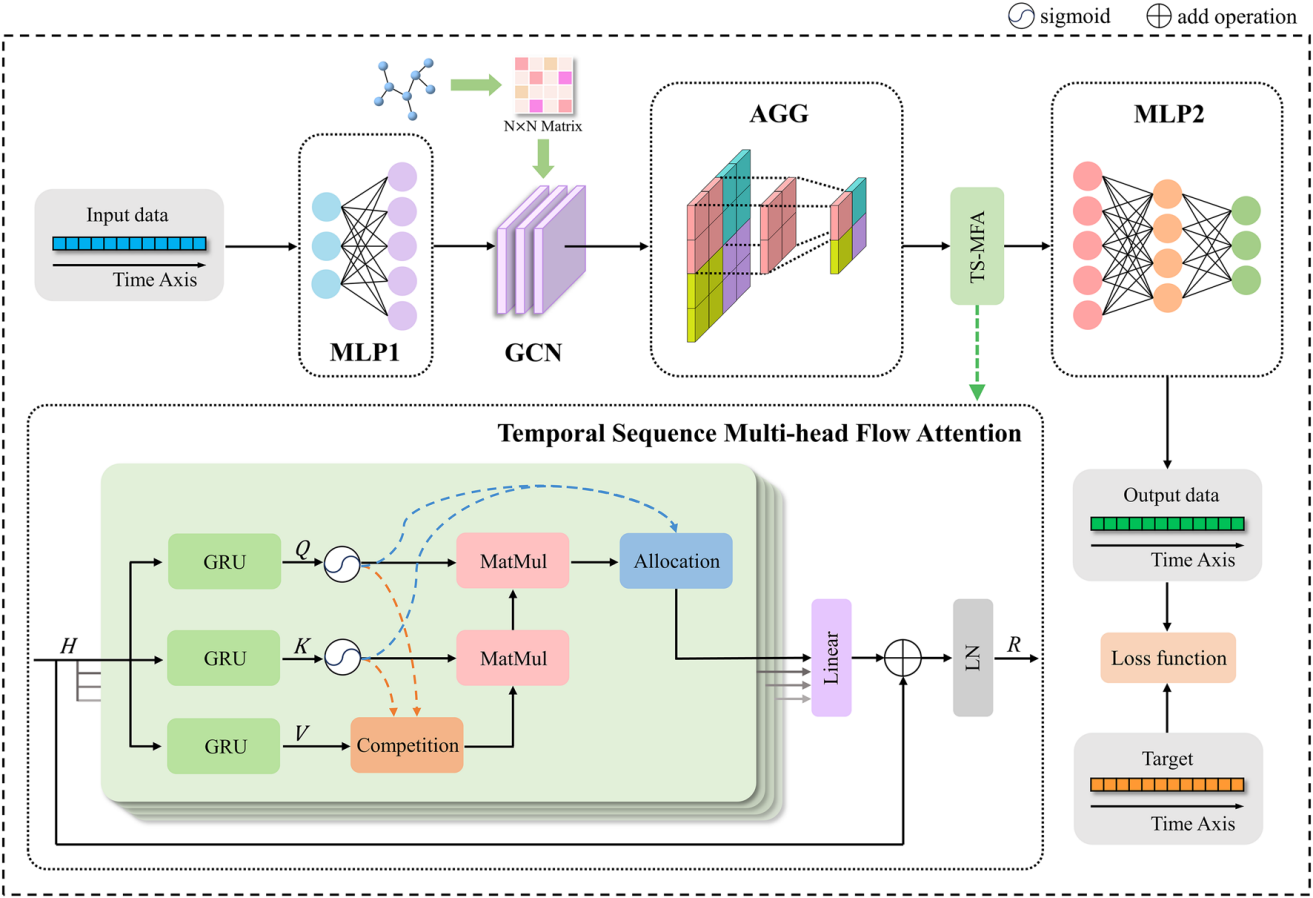
The overall structure of STCMFA.

Figure 3 shows the overall framework of STCMFA, which is mainly composed of input layer (Multi-Layer Perceptron, MLP1), GCN, aggregation layer, TS-MFA module, and output layer (MLP2). We will describe each module of STCMFA in detail below.

#### Graph convolutional network

The spectral graph theory studies the influence of the eigenvalues and corresponding eigenvectors of the Laplacian matrix of the graph on the topological properties of the graph^42^. In spectral analysis, for a graph $G$, its Laplacian matrix can be expressed as:

2 $$\begin{array}{c}L={I}_{N}-{D}^{-\frac{1}{2}}A{D}^{-\frac{1}{2}},\end{array}$$

where $A$ is an adjacency matrix, and the degree matrix $D\in {R}^{N\times N}$ is a diagonal matrix, defined as ${D}_{ii}=\sum_{j}{A}_{ij}$. ${I}_{N}$ is a unit matrix of size $N\times N$, $L\in {R}^{N\times N}$. The eigenvalue decomposition of the Laplacian matrix is obtained:

3 $$\begin{array}{c}L=U\Lambda {U}^{T},\end{array}$$

4 $$\begin{array}{c}\Lambda =diag\left(\left[{\lambda }_{0},{\lambda }_{1},\dots ,{\lambda }_{N-1}\right]\right)\in {R}^{N\times N}.\end{array}$$

In Eq. (3), $\Lambda$ is a diagonal matrix composed of eigenvalues of $L$, as shown in Eq. (4). $U=\left\{{u}_{1},{u}_{2},\dots ,{u}_{N}\right\}$ is the eigenvector of $L$.

We set $x={X}_{t}\in {R}^{N}$ as the signal of the whole graph, and define the Fourier transform of the signal as $\widehat{x}={U}^{T}x$. The signal $x$ on $G$ can be filtered by the convolution kernel ${g}_{\theta }$:

5 $$\begin{array}{c}{g}_{\theta }*Gx=U{g}_{\theta }\left(\Lambda \right){U}^{T}x,\end{array}$$

where $*G$ denotes the graph convolutional operation. To speed up the solution of the characteristic matrix, we use the Chebyshev polynomial to approximate the eigenvalue matrix. The Chebyshev polynomials can be expressed by Eq. (6):

6 $$\begin{array}{c}{g}_{\theta }\left(\Lambda \right)=\sum_{k=0}^{K-1}{\theta }_{k}{T}_{k}\left(\widetilde{\Lambda }\right),\end{array}$$

where $\theta \in {R}^{K}$ is the vector of Chebyshev polynomial coefficients, and ${T}_{k}\left(\widetilde{\Lambda }\right)\in {R}^{N\times N}$ is the Chebyshev polynomial of order $K$ of $\widetilde{\Lambda }$. And $\widetilde{\Lambda }=\frac{2}{{\lambda }_{max}}\Lambda -{I}_{N}$, that is, the eigenvalue diagonal matrix after the range normalization, ${\lambda }_{max}$ is the maximum value of the eigenvalue. The recurrence formula of Chebyshev polynomials of order $k$ is ${T}_{k}=2x{T}_{k-1}\left(x\right)-{T}_{k-2}\left(x\right)$. The first two terms of the recurrence equation are ${T}_{0}\left(x\right)=1, {T}_{1}\left(x\right)=x$. The graph convolutional operation after Chebyshev approximation can be defined as the following equation:

7 $$\begin{array}{c}{g}_{\theta }*Gx=U{g}_{\theta }\left(\Lambda \right){U}^{T}x=U\left(\sum_{k=0}^{K-1}{\theta }_{k}{T}_{k}\left(\widetilde{\Lambda }\right)\right){U}^{T}x\approx \sum_{k=0}^{K-1}{\theta }_{k}{T}_{k}\left(\widetilde{L}\right)x,\end{array}$$

where $\widetilde{L}=\frac{2}{{\lambda }_{max}}L-{I}_{N}$. Equation (7) can be understood as extracting the information of 0 to $K-1$ neighbors around each node in the topology graph through the convolution kernel ${g}_{\theta }$. After each graph convolutional operation, we use the Rectified Linear Unit (ReLU) as the activation function to activate, that is, ReLU (${g}_{\theta }*Gx$).

#### Temporal correlation modeling

There are many methods that can model temporal correlation, such as one-dimensional convolutional neural network (1D-CNN), RNN, LSTM, and GRU. However, the convolution operation of 1D-CNN only focuses on local feature information, and its ability to model long-term dependencies in sequences is limited. Due to the special cyclic structure and back propagation algorithm of RNN, it has the exploding and the vanishing gradient problems when dealing with long sequences. Therefore, the variants of RNN, LSTM and GRU, have been proposed one after another. Compared with the LSTM with complex structure and slow training speed, GRU has relatively simple structure, less parameters, and fast training speed. In addition, its special gating mechanism can flexibly control the flow of information, and has the ability to capture long-term dependencies. Therefore, we use GRU to capture the temporal correlation of traffic flow. The specific calculation process of GRU is as follows:

8 $$\begin{array}{c}{z}_{t}=\sigma \left({W}_{z}{x}_{t}+{U}_{z}{h}_{t-1}+{b}_{z}\right),\end{array}$$

9 $$\begin{array}{c}{r}_{t}=\sigma \left({W}_{r}{x}_{t}+{U}_{r}{h}_{t-1}+{b}_{r}\right),\end{array}$$

10 $$\begin{array}{c}{\widetilde{h}}_{t}=tanh\left({W}_{h}{x}_{t}+{U}_{h}\left({{r}_{t}\otimes h}_{t-1}\right)+{b}_{h}\right),\end{array}$$

11 $$\begin{array}{c}{h}_{t}=\left(1-{z}_{t}\right)\otimes {h}_{t-1}+{z}_{t}\otimes {\widetilde{h}}_{t},\end{array}$$

where ${W}_{z}, {W}_{r}, {W}_{h}$ and ${U}_{z}, {U}_{r}, {U}_{h}$ are weight matrices, ${b}_{z}, {b}_{r}, {b}_{h}$ are biases, and $\sigma$ is a sigmoid function. Let $x=\left\{\dots ,{x}_{t-1},{x}_{t},{x}_{t+1},\dots \right\}$ denote the continuous sequence information of a node in the data set, ${x}_{t}$ is the input at time t, ${h}_{t-1}$ is the hidden state of the output at the previous time, ${\widetilde{h}}_{t}$ is the candidate hidden state at the current time, ${h}_{t}$ is the hidden state of the output at the current time, $\otimes$ is the Hadamard product. ${z}_{t}$ and ${r}_{t}$ represent the update gate and reset gate respectively. The update gate controls the retention degree of new and old information, and the reset gate controls the retention degree of input at the previous moment.

#### Temporal sequence multi-head flow attention

The attention mechanism can be described as a mapping mechanism between the query vector $\left(Q\right)$ and a series of key-value vector pairs $\left(K,V\right)$ and the output vector, where the weighting coefficients of each value vector are obtained by the scaled dot product of the query vector and the key vector, and the output vector is obtained by the weighted sum of the value vectors.

The traditional attention mechanism needs to calculate the similarity between the query at each location and the keys at all locations, which leads to a quadratic increase in the amount of computation required to calculate the attention weight when dealing with long sequences. The flow attention mechanism introduces the idea of flow conservation into both source and sink, and proposes a source competition mechanism and a sink allocation mechanism to achieve competition between tokens without using inductive biases. Flow attention can effectively solve the quadratic complexity problem caused by similarity calculation in traditional attention.

A single attention mechanism often only establishes a dependency between the query vector and the key vector, which leads to its limited modeling ability. We use multi-head flow attention to learn different dependencies from the same attention aggregation. Like traditional attention, flow attention obtains $Q$, $K$, and $V$ by linear transformation through the fully connected layer, which limits its ability to model temporal sequence data. To further enhance the temporal modeling ability of the model, we propose a temporal sequence multi-head flow attention (TS-MFA) using GRU to map input data, as shown in Fig. 3.

To achieve the competition between information, the flow attention mechanism introduces conservation properties in both the source (the process of obtaining information from the previous layer) and the sink (the process of providing information to the next layer). Specifically, by setting the incoming flow of each sink to 1, the outgoing flow of the source will compete with each other because of the only location. Similarly, by setting the source outgoing flow to 1, the sink will compete for the only flow. After obtaining $Q$, $K$, and $V$ through GRU mapping, we achieve the conservation of the above two aspects of source and sink by performing a normalization operation on $Q$ and $K$ respectively. Suppose there are $n$ sinks and $m$ sources, the two normalization processes are shown in Eqs. (12) and (13).

12 $$\begin{array}{c}\frac{\varphi \left(Q\right)}{I},\end{array}$$

13 $$\begin{array}{c}\frac{\varphi \left(K\right)}{O},\end{array}$$

where $\varphi$ denotes a nonnegative nonlinear function, $I\in {R}^{n\times 1}$ and $O\in {R}^{m\times 1}$ represent the incoming flow of sink and the outgoing flow of source, respectively. $\frac{\varphi \left(Q\right)}{I}$ denotes the sink conservation, $\frac{\varphi \left(K\right)}{O}$ denotes the source conservation.

Through standardization, the source outgoing flow conservation and sink incoming flow conservation are realized. The specific calculation process is as shown in Eqs. (14) and (15).

14 $$\begin{array}{c}{I}{\prime}=\varphi \left(Q\right)\sum_{j=1}^{m}\frac{\varphi {\left({K}_{j}\right)}^{T}}{{O}_{j}},\end{array}$$

15 $$\begin{array}{c}{O}{\prime}=\varphi \left(K\right)\sum_{i=1}^{n}\frac{\varphi {\left({Q}_{i}\right)}^{T}}{{I}_{i}},\end{array}$$

where $i\in \left\{1, 2, \dots ,n\right\}$ and $j\in \left\{1, 2,\dots ,m\right\}$, $I{\prime}\in {R}^{n\times 1}$ and $O{\prime}\in {R}^{m\times 1}$ represent the amount of information of the conserved incoming flow and outgoing flow, respectively.

TS-MFA mainly includes three parts: competition, aggregation, and allocation, the equations are as follows:

16 $$ \begin{array}{*{20}c} {Competition: V^{\prime} = softmax\left( {O^{\prime}} \right) \odot V,} \\ \end{array} $$

17 $$\begin{array}{c}Aggregation: A=\frac{\varphi \left(Q\right)}{I}\left(\varphi {\left(K\right)}^{T}{V}{\prime}\right),\end{array}$$

18 $$ \begin{array}{*{20}c} {Allocation: R = LayerNorm\left( {sigmoid\left( {I^{\prime}} \right) \odot A + H} \right),} \\ \end{array} $$

where $\odot$ denotes the element-wise multiplication, $H$ represents the input of the TS-MFA module. Competition is achieved by nontrivial reweighting based on input flow conservation. Information aggregation is carried out according to the associativity of matrix multiplication. The allocation mechanism uses ${I}{\prime}$ to filter the incoming flow of each sink to obtain the final output of the TS-MFA module. We integrate the residual mechanism to solve the problem of network degradation to a certain extent, and reduce the risk of gradient disappearance through layer normalization.

#### Extra components

##### Input layer

We use a fully connected layer as the input layer of the model, which can convert low-dimensional input data into higher dimensions, thereby improving the expression ability and prediction accuracy of the model.

##### Aggregating layer

As shown in Fig. 3, AGG represents the aggregation layer, and we use max pooling as the specific method of aggregation. The output data of the graph convolutional networks is subjected to a one-dimensional max pooling operation in the time dimension, that is, an element with the largest value is selected in each sliding window, and the max pooling sliding window size is set to 2.

##### Output layer

The output of the TS-MFA module passes through the output layer MLP2 to obtain the final output of the STCMFA model.

##### Loss function

We use MAE as the loss function.

The training process of the model is shown in Algorithm 1.

**Algorithm 1:**
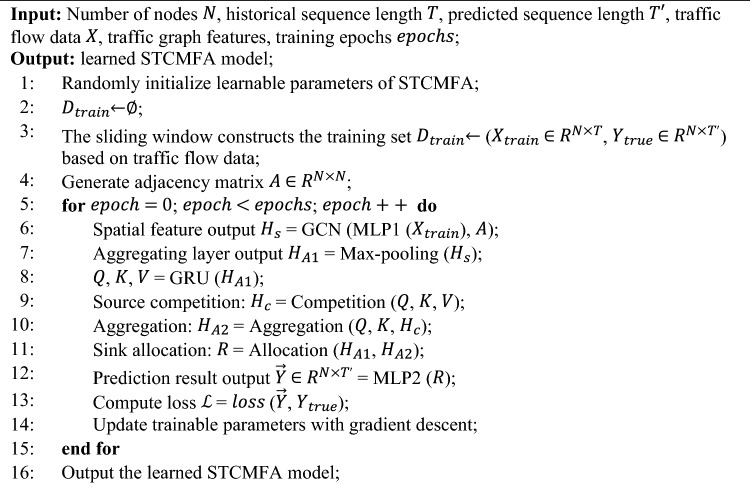
Training algorithm of STCMFA.

## Experiments

### Datasets

To fully prove the prediction performance and generalization ability of the proposed model, we conduct extensive experiments on four real-world traffic datasets: PeMS03, PeMS04, PeMS07, and PeMS08^8^. These datasets are collected by the Caltrans performance measurement system (PeMS) on highways in California, which deployed more than 39,000 sensors on some major highways in California. The system collects data in real time every 30 s. Once the data set of 30 s is compiled, the original data will be aggregated into a 5-min interval without any gap. That is, there are 288 time steps in the data collected by each sensor in 1 day.

The original data of the PeMS series include three indicators: traffic flow, speed, and occupancy. In this paper, we only use traffic flow data for traffic prediction research. The details of these datasets are shown in Table 1, where nodes is the number of road sensors, time steps is the complete time steps of the data collected by each sensor, edges is the number of connections between sensors. The spatial connection relationship of each dataset is constructed based on the actual road connection network. If two sensors are on the same road, we think they are spatially connected.

**Table 1:** Dataset description.

Datasets	Nodes	Days	Time steps	Time range	Edges
PeMS03	358	91	26,208	9/1/2018–11/30/2018	547
PeMS04	307	59	16,992	1/1/2018–2/28/2018	340
PeMS07	883	98	28,224	5/1/2017–8/31/2017	866
PeMS08	170	62	17,856	7/1/2016–8/31/2016	295

### Experiment settings

To be fair, we maintain the same data partitioning method as other baselines, and divide all datasets into training set, validation set, and test set according to the ratio of 6:2:2. We use one hour of historical data to predict traffic flow in the next hour, that is, we use the past 12 continuous time steps to predict the next 12 continuous time steps.

All experiments are conducted on a server with NVIDIA GeForce RTX3090 GPU. We use one layer of GRU, and its number of hidden units is 64. The number of terms of the Chebyshev polynomial is K = 3, and the number of attention heads in the attention module is 4. Our model contains three graph convolutional operations. We use the Adam optimizer to train our model with a learning rate of 0.001. The batch size is 16.

We use the following three indicators to evaluate the performance of the model:

19 $$\begin{array}{c}MAE=\frac{1}{N\times T}\sum_{t=1}^{T}\sum_{n=1}^{N}\left|{Y}_{t}^{n}-{\widehat{Y}}_{t}^{n}\right|,\end{array}$$

20 $$\begin{array}{c}MAPE=\frac{1}{N\times T}\sum_{t=1}^{T}\sum_{n=1}^{N}\frac{\left|{Y}_{t}^{n}-{\widehat{Y}}_{t}^{n}\right|}{{Y}_{t}^{n}},\end{array}$$

21 $$\begin{array}{c}RMSE=\sqrt{\frac{1}{N\times T}\sum_{t=1}^{T}\sum_{n=1}^{N}{\left({Y}_{t}^{n}-{\widehat{Y}}_{t}^{n}\right)}^{2}}.\end{array}$$

### Baseline methods

We compare STCMFA with following baseline models:

1. LSTM^7^: Long short-term memory network is used to capture the correlation of traffic data in the time dimension.
2. DCRNN^27^: The traffic flow is modelled as a diffusion process on a directed graph, and the spatial dependence is captured by bidirectional random walk on the graph, and the temporal correlation is captured by using the seq2seq architecture with predetermined sampling.
3. STGCN^28^: STGCN combines graph convolution and 2D convolution to effectively capture comprehensive spatial–temporal correlation.
4. ASTGCN(r)^37^: ASTGCN models three time attributes of recent, daily, and weekly periodicity respectively, and uses the spatial–temporal attention mechanism to effectively capture the dynamic spatial–temporal correlation in traffic flow data. To be fair, we only use it to model the latest component of periodicity (ASTGCN(r)).
5. STG2Seq^43^: Combining the graph volume product with attention mechanism and seq2seq to capture the temporal-spatial correlation.
6. Graph WaveNet^29^: An adaptive adjacency matrix is proposed to capture the hidden spatial dependence, and the graph convolution and dilated casual convolution are combined to capture the spatial–temporal correlation.
7. STSGCN^8^: Spatial–temporal synchronous graph convolutional networks uses multiple local spatial–temporal subgraph modules to synchronously capture the heterogeneity in the local spatial–temporal graph.
8. STGODE^9^: Spatial–temporal graph ODE networks captures spatial–temporal dynamics through tensor-based ordinary differential equations (ODE) and captures long-term temporal correlation based on semantic adjacency matrix.
9. STDSGNN^10^: Spatial–temporal dynamic semantic graph neural network captures semantic features by constructing two semantic adjacency matrices, and uses multi-head graph attention and full convolution to capture spatial correlation and temporal correlation respectively.
10. STGPCN(Kronecker)^32^: A general framework for modeling dynamic spatial–temporal graphs is constructed, and a spatial–temporal adjacency graph construction method is proposed to increase the spatial–temporal receptive field.
11. LEISN-ED^44^: A long-term explicit–implicit spatial–temporal network is proposed, which promotes the transmission of long-term features through a long-term dependency module, and two spatial feature extraction branches are designed to extract explicit and implicit spatial features respectively.
12. STSGRU^11^: A spatial–temporal shared GRU is proposed, which captures long-term temporal features by modeling weekly traffic patterns, and a shared weight mechanism is designed to achieve many-to-many prediction.

### Experiment results and analysis

#### Results on PeMS series datasets

Table 2 shows the traffic flow prediction results of STCMFA and baseline models. The bold marker represents the best indicator in all results, “–” represents the corresponding baseline uncalculated indicator. Overall, the prediction results of our STCMFA model on four datasets are significantly better than all baseline models.

**Table 2:** Comparison of traffic flow prediction performance between STCMFA and baseline models.

Model	PeMS03			PeMS04			PeMS07			PeMS08		
	MAE	MAPE (%)	RMSE	MAE	MAPE (%)	RMSE	MAE	MAPE (%)	RMSE	MAE	MAPE (%)	RMSE
LSTM	21.33	23.33	35.11	27.14	18.20	41.59	29.98	13.20	45.84	22.20	14.20	34.06
DCRNN	18.18	18.91	30.31	24.70	17.12	38.12	25.30	11.66	38.58	17.86	11.45	27.83
STGCN	17.49	17.15	30.12	22.70	14.59	35.55	25.38	11.08	38.78	18.02	11.40	27.83
ASTGCN(r)	17.69	19.40	29.66	22.93	16.56	35.22	28.05	13.92	42.57	18.61	13.08	28.16
STG2Seq	19.03	21.55	29.73	25.20	18.77	38.48	32.77	20.16	47.16	20.17	17.32	30.71
Graph WaveNet	19.85	19.31	32.94	25.45	17.29	39.70	26.85	12.12	42.78	19.13	12.68	31.05
STSGCN	17.48	16.78	29.21	21.19	13.90	33.65	24.26	10.21	39.03	17.13	10.96	26.80
STGODE	16.50	16.69	27.84	20.84	13.77	32.82	22.99	10.14	37.54	16.81	10.62	25.97
STDSGNN	16.12	16.15	25.59	20.67	13.83	32.40	22.91	10.06	34.95	16.73	10.84	25.59
STGPCN (Kronecker)	17.11	16.48	28.99	20.96	13.78	33.35	24.02	10.08	38.77	16.41	10.43	25.60
LEISN-ED	15.83	14.66	26.05	–	–	–	–	–	–	15.94	10.18	24.96
STSGRU	**15.45**	15.85	24.13	20.11	13.86	31.80	21.50	9.08	34.40	**15.68**	10.67	25.12
STCMFA(our)	15.48	**13.52**	**22.91**	**19.78**	**12.51**	**29.51**	**21.34**	**8.39**	**33.39**	16.09	**9.27**	**24.12**

Specifically, LSTM as a temporal sequence model can only capture the temporal correlation in traffic data, and does not have the ability to capture spatial dependence. Other baselines can capture both temporal correlation and spatial dependence at the same time, so LSTM has the worst effect in all baseline models. In contrast, baseline models such as Graph WaveNet and STGCN both take into account the influence of time and space on traffic flow changes, so they achieve better prediction results. However, the time and space modeling modules in these models are very basic and simple, so their prediction performance still has great limitations.

STSGCN uses multiple local spatial–temporal subgraphs to capture the heterogeneity of spatial–temporal data, which can better mine the interaction between spatial–temporal data. STGODE combines tensor-based ordinary differential equations to extract long-term spatial–temporal correlation. Compared with the local spatial–temporal correlation captured by STSGCN, STGODE has greater advantages in global dependence. STDSGNN, STGPCN, LEISN-ED, and STSGRU reported recently have further improved the ability to model spatial–temporal correlation through feature construction and feature extraction, and achieved better prediction results. Among them, the prediction result of STSGRU is the best, which improves the flexibility and performance of the model by modeling periodicity and designing a shared weight mechanism.

Compared with STSGRU, the prediction results of STCMFA are significantly better. This is because STCMFA captures the key information in the sequence by introducing the flow attention mechanism, and avoids the calculation of attention weight, which effectively reduces the complexity of the model. In addition, the temporal sequence model is integrated into the flow attention and combined with the graph convolutional module, which greatly enhances the ability of the model to capture long-term dependency and spatial–temporal correlation, and improves the accuracy of traffic flow prediction.

#### Visualization

**Figure 4:**
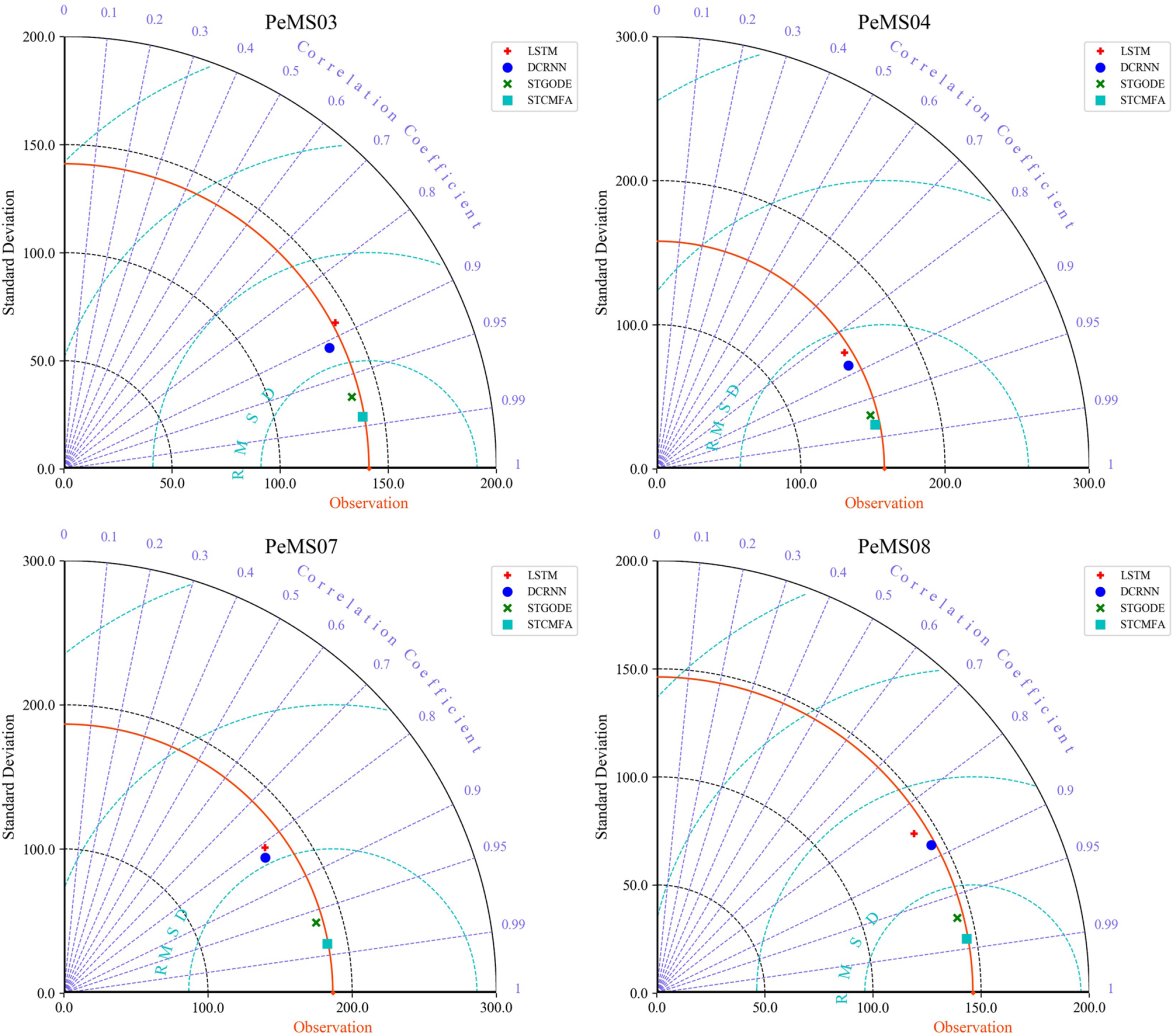
The Taylor diagrams on four datasets.

To compare the performance of the model more intuitively, we draw the Taylor diagram of the STCMFA and baseline models, as shown in Fig. 4. The correlation coefficient of the proposed model is significantly higher and its standard deviation is closer to the observation, indicating that its prediction performance is the best.

**Figure 5:**
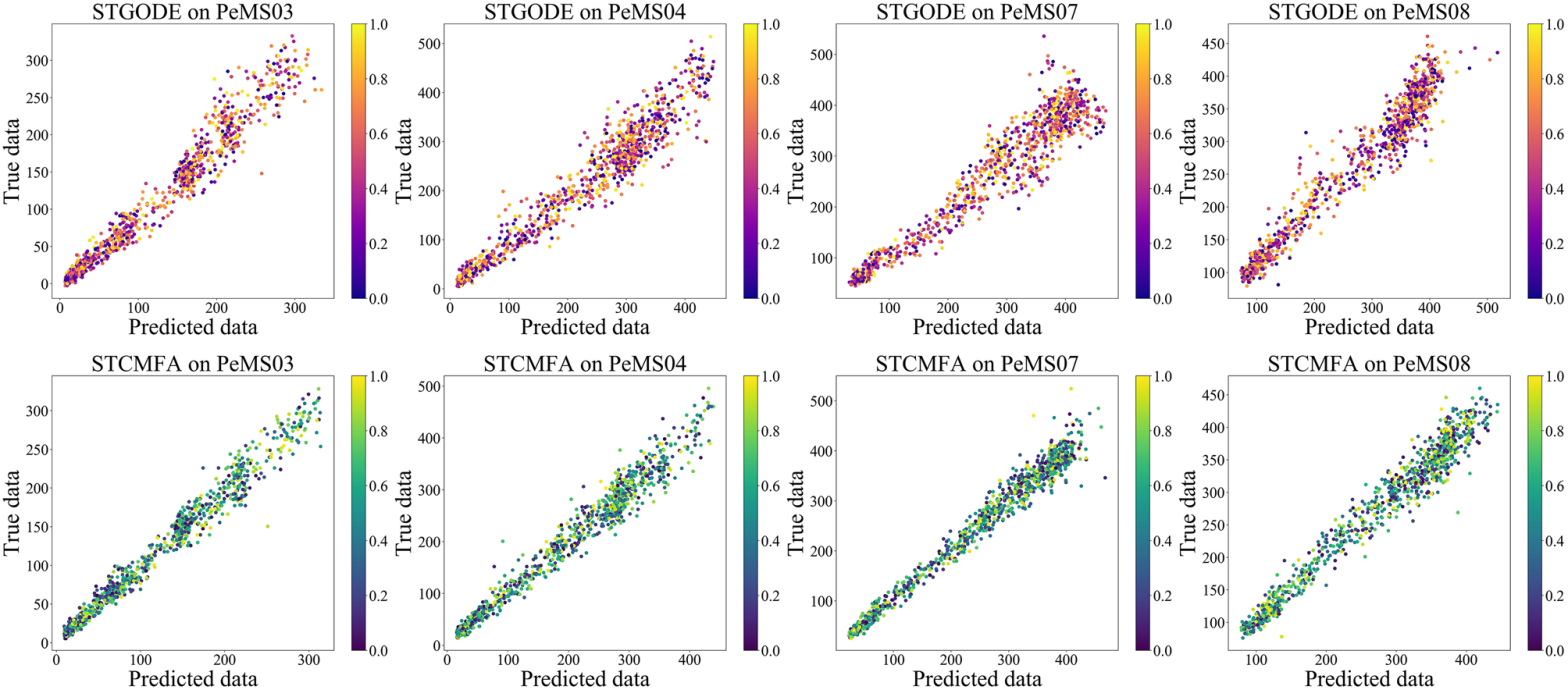
The scatter plot of STCMFA and STGODE.

Figure 5 is the scatter plot of STCMFA and STGODE on four datasets. The horizontal axis and the vertical axis are the predicted value and the true value, respectively. It can be seen that the scatter plot of the proposed model is more aggregated, indicating that its prediction accuracy is higher than that of STGODE.

**Figure 6:**
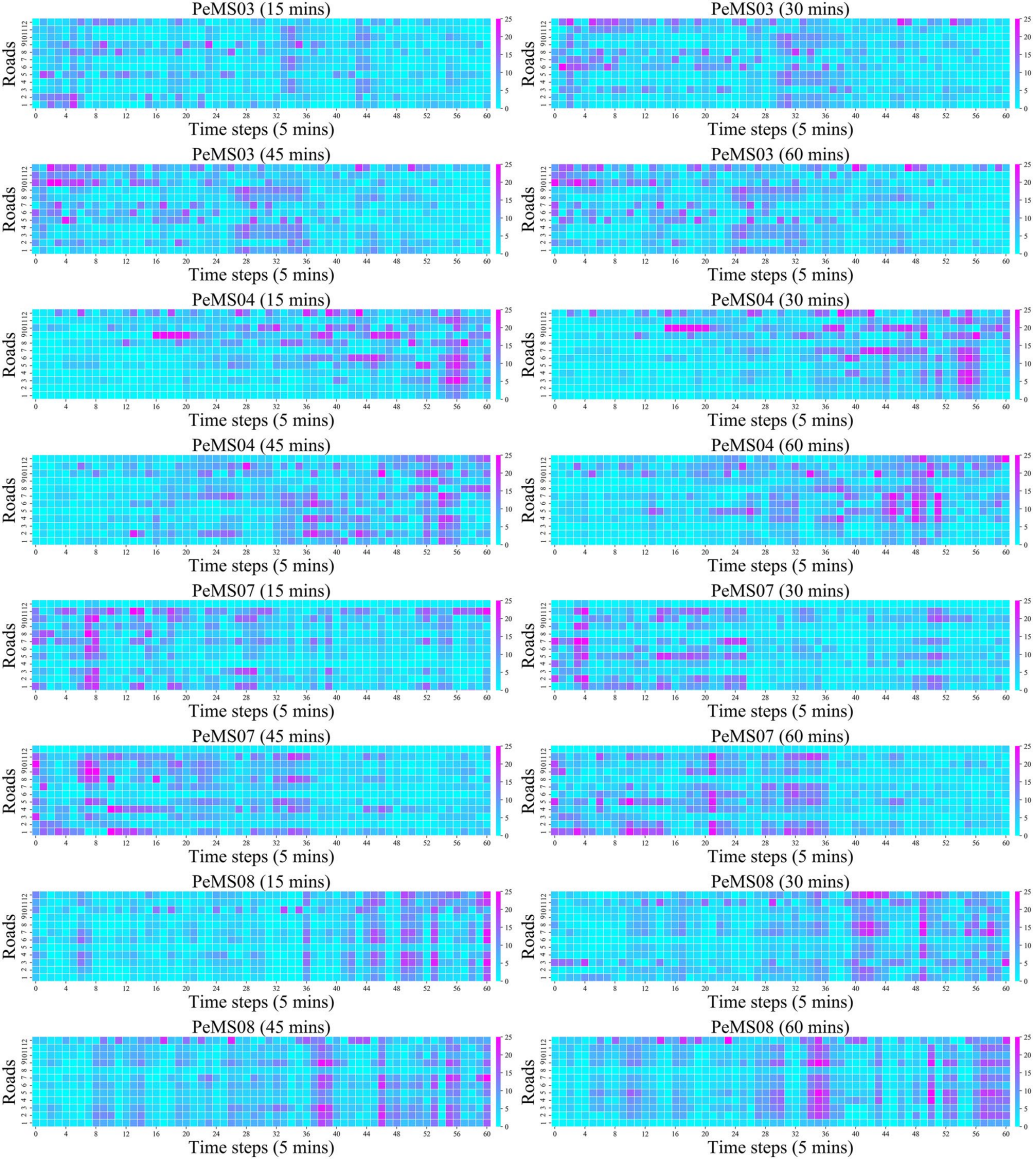
Heatmaps on different prediction horizons.

Figure 6 shows the absolute error heatmaps of the predicted values and true values of the STCMFA model on four datasets. Due to large datasets, we selected the first 60 time steps of 12 roads in each dataset for display, and drew four different prediction horizon heatmaps on each dataset. Usually, as the prediction horizon increases, the prediction performance of the model will gradually decrease. However, we can find from the heatmaps that STCMFA has good results in both short-term and long-term prediction horizons. This is due to the fact that we have improved the flow attention mechanism based on GRU. The special gating mechanism in GRU can flexibly control the transmission of feature information, enhance the ability of modeling long-term dependence, and improve the prediction effect of long horizons.

**Figure 7:**
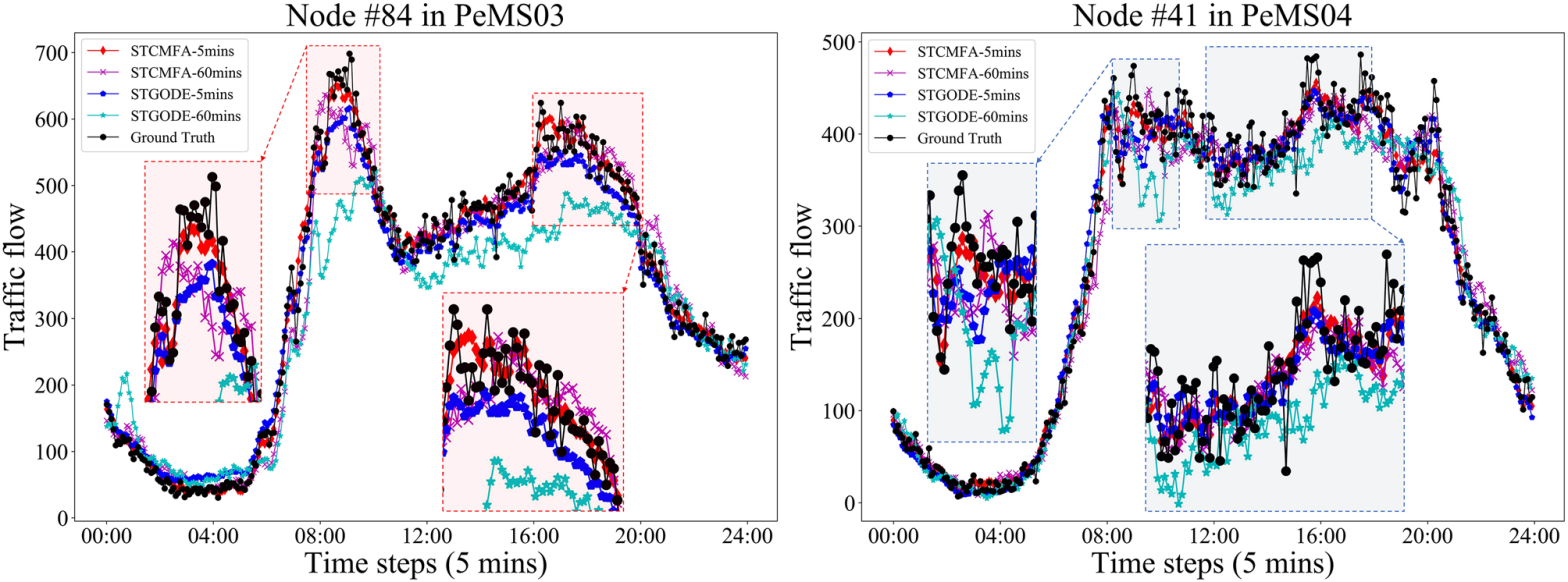
Comparison of prediction curves between STCMFA and STGODE.

**Figure 8:**
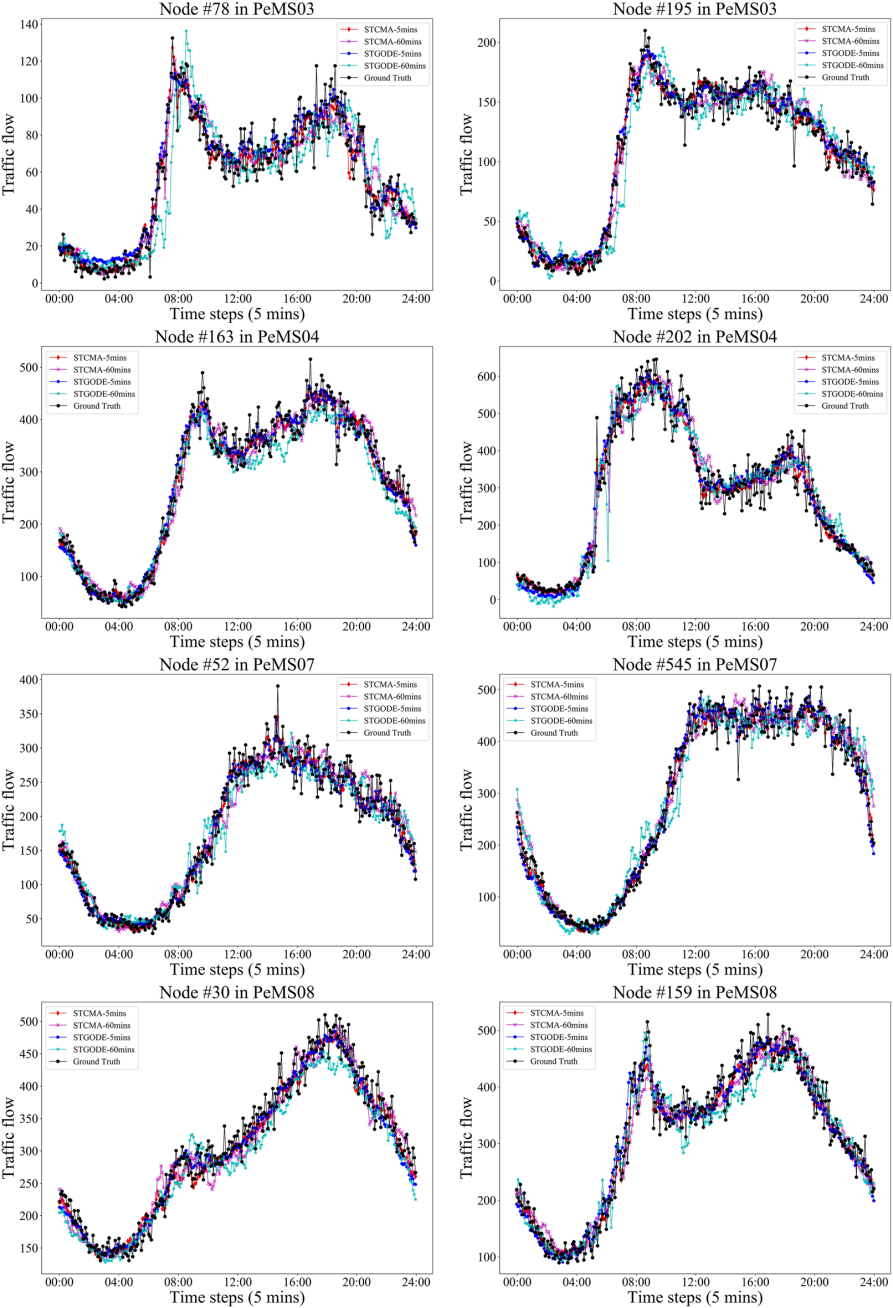
The prediction curves of four datasets.

In Fig. 7, we cut out the time period of one day on the test set of PeMS03 and PeMS04, and then draw the 5-min and 60-min prediction curves of STCMFA and STGODE respectively, and compare them with the ground truth. From the red dotted line box in Fig. 7, it can be found that when the traffic data suddenly rises or falls, our STCMFA predicts this change more sensitively and quickly than STGODE, and the predicted values are more accurate. This is because STCMFA integrates the temporal sequence model into the flow attention mechanism, so that the model can pay attention to the special change patterns of the sequence in the time dimension, so as to predict these changes more quickly and accurately. As shown in the blue dotted box in Fig. 7, STCMFA not only performs well in the short-term prediction of 5 min, but also has a better effect than STGODE in the long-term prediction of 60 min. Figure 8 shows the prediction curves for all datasets.

**Figure 9:**
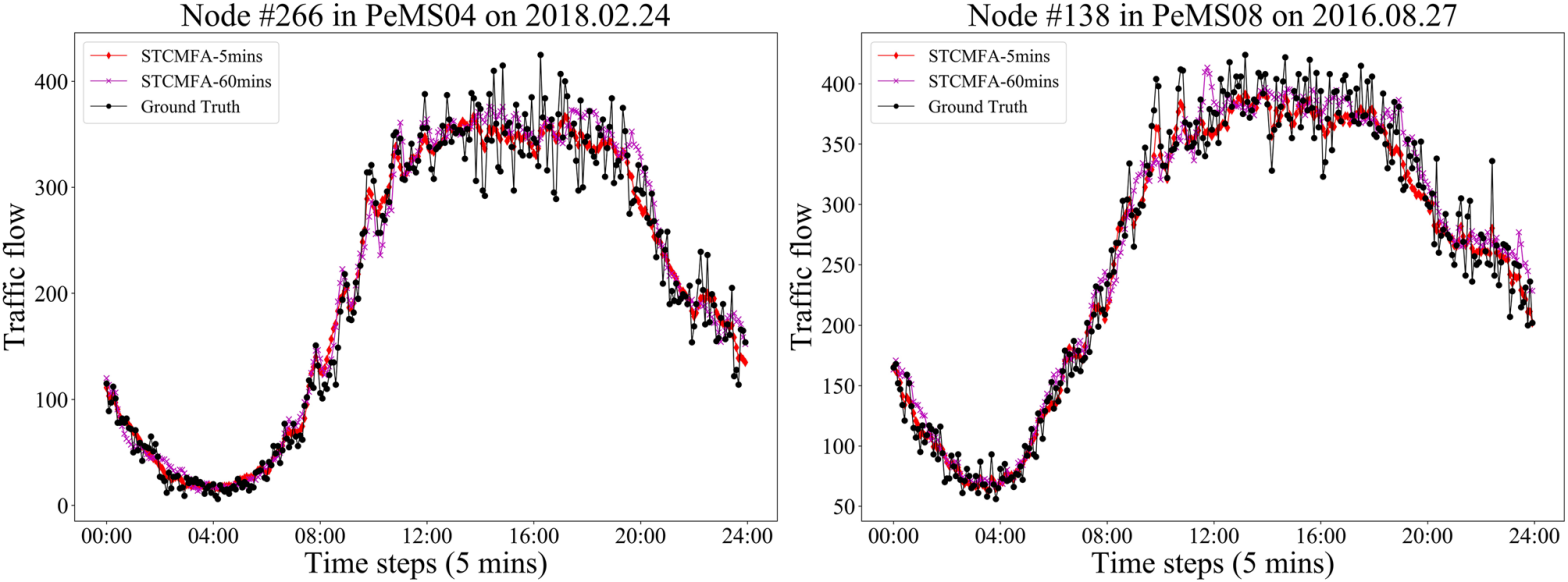
The prediction curves of weekend time period.

In addition, we cut out the time period of PeMS04 and PeMS08 on the weekend day, and draw the 5-min and 60-min prediction curves of STCMFA, as shown in Fig. 9. It can be found that the traffic flow curve during the working day in Fig. 8 usually has two peaks due to commuting, while the traffic flow during the weekend in Fig. 9 has remained at a high level during the day, which is very consistent with the reality. From Fig. 9, it can also be found that STCMFA also has a good fitting effect in the special time period of the weekend. This is due to the special multi-head flow attention mechanism in the model. The multi-head parallel learning of different feature information in multiple subspaces enables the model to accurately predict different types of sequence changes, and enhances the generalization ability of the model.

### Ablation analysis

Ablation experiments mainly evaluate the degree of influence on the function of the whole system by systematically stripping or changing a factor. In traffic flow prediction, ablation experiments are usually used to determine the role and contribution of specific modules in the entire model. Therefore, to verify the effectiveness of different modules of STCMFA, we design four variants of the STCMFA model. All these model variants are tested under the best parameters. We compare the prediction results of these four variants and STCMFA on PeMS04 and PeMS08 datasets.

1. *Only-spatial* Only spatial graph convolution network is used to capture the dependence of spatial information without considering the correlation of time series, so as to verify the necessity of spatial–temporal combination.
2. *REPL-FAtt* Replace the flow attention mechanism with traditional attention to prove the advantages of flow attention.
3. *RM-TS* The temporal sequence module of TS-MFA is removed to verify the necessity of enhancing the ability of flow attention to capture temporal correlation.
4. *REPL-Agg* Remove the max pooling aggregation layer and use the ordinary fully connected layer to reduce the dimension.

**Figure 10:**
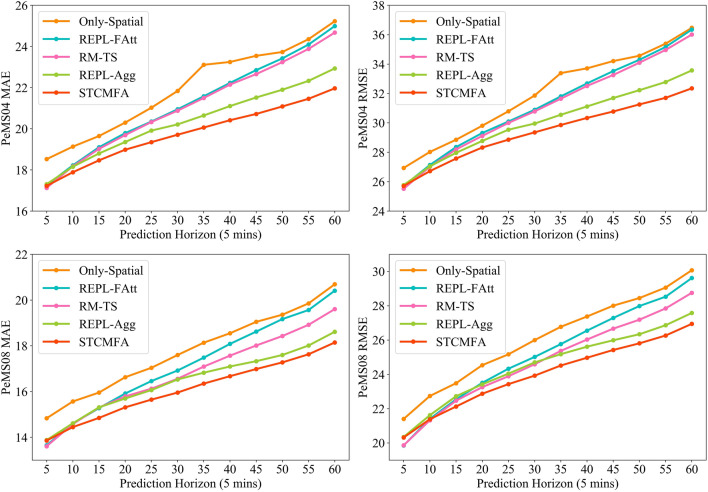
The results of ablation experiments on each prediction horizon.

Figure 10 shows the results of the ablation experiment. On the whole, the prediction effect of STCMFA is significantly better than that of the four variant models. This proves the effectiveness of each key module in STCMFA, which plays an active role in improving the prediction accuracy of the model.

Specifically, the prediction effect of the only-spatial variant is the worst, because it only considers spatial dependence and ignores temporal correlation. This shows that it is necessary to capture the spatial–temporal correlation in traffic sequences. After replacing the flow attention with ordinary attention, the prediction effect becomes worse, which indicates the superiority of the unique competition and allocation mechanism within the flow attention mechanism. In addition, we calculated the model parameters of REPL-FAtt and STCMFA, which are 2,874,254 and 2,184,472, respectively. Under the condition that the other parts of the model remain unchanged, only replacing traditional attention with flow attention significantly reduces the number of model parameters, which shows that flow attention can indeed solve the quadratic complexity problem of traditional attention. We integrate the temporal sequence model into the flow attention to effectively enhance the temporal sequence modeling ability of the flow attention. Compared with the use of ordinary full connection layer to reduce the dimension, the effect of selecting the max pooling as the aggregation operation is obviously better. This is because max pooling can reduce the dimension while retaining the salient features to the maximum extent, which can improve the training speed and prediction performance of the model.

### Effect of hyperparameters

**Figure 11:**
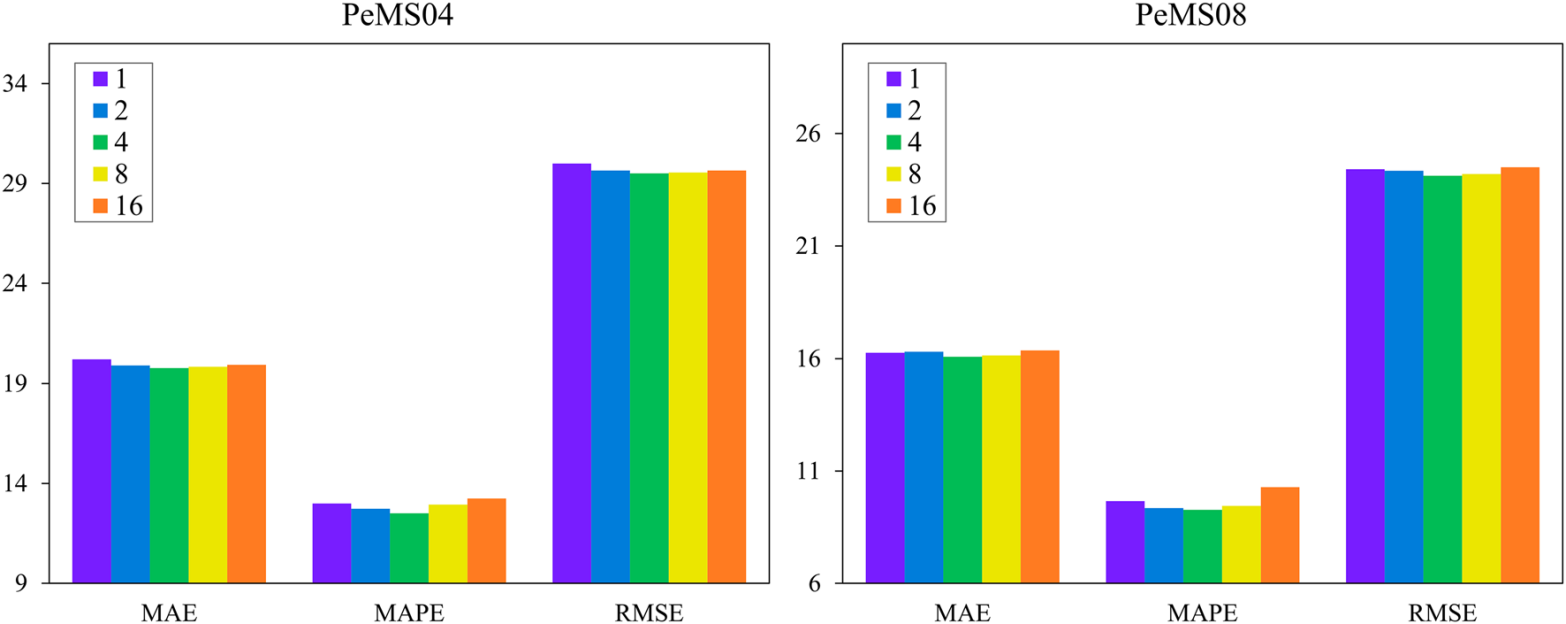
Effects of different attention heads.

Setting different hyperparameters in the same model will have a certain impact on the prediction effect of the model. As shown in Fig. 11, we set different attention heads on PeMS04 and PeMS08 respectively and compare their prediction results. It can be found that appropriately increasing the number of attention heads will improve the prediction effect of the model, but if too much is added, the model will lead to a decline in its prediction performance due to overfitting.

### Training process and cost

**Figure 12:**
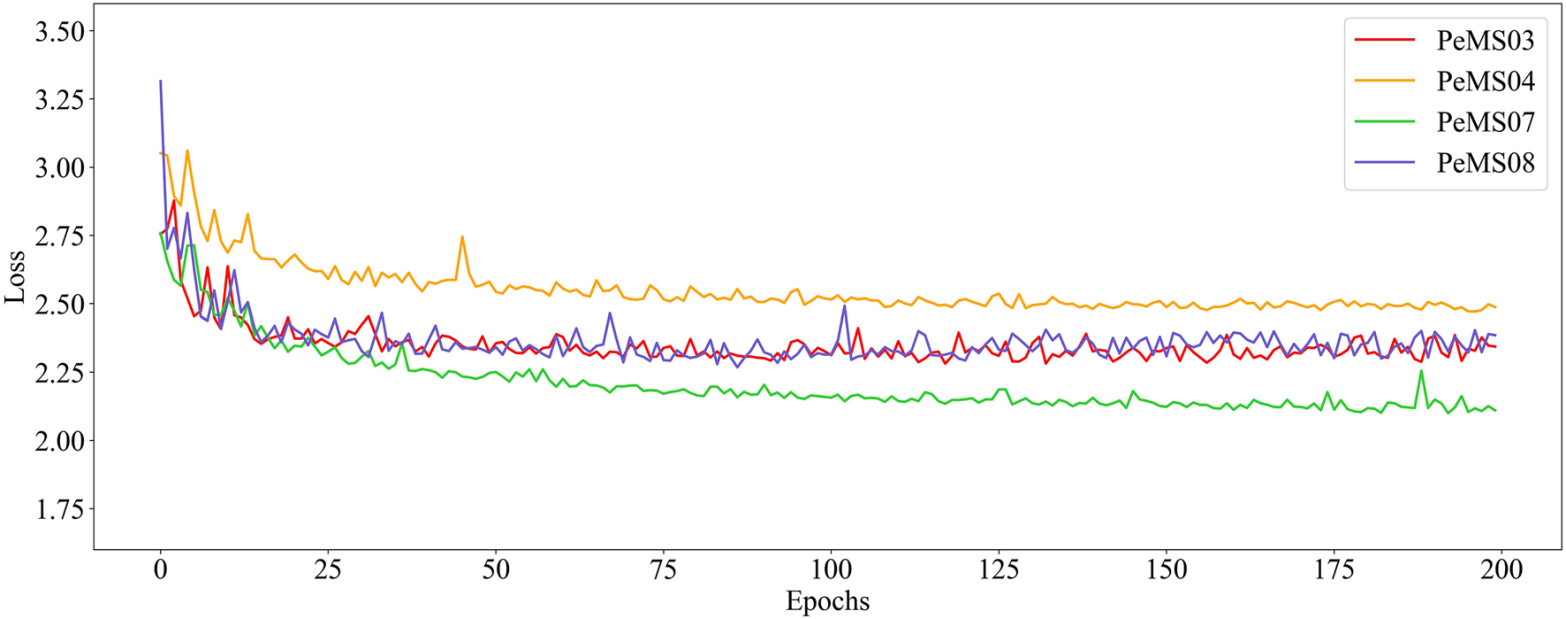
The loss changes of STCMFA on four datasets.

Figure 12 shows the change of STCMFA loss during training. With the increase of training epochs, the loss of STCMFA on each dataset shows a gradual downward trend. When the loss decreases to a certain extent, it gradually converges to a smooth state.

Table 3 shows the time consumption and memory usage of STCMFA on four datasets. Obviously, in the case of the same batch size, the larger the dataset, the higher the time and memory consumption. The internal competition mechanism of the proposed model avoids the calculation of attention weight, reduces the complexity of the model to a certain extent, and accelerates the training speed of the model.

**Table 3:** Time and memory consumption on four datasets.

Datasets	Training (s/epoch)	Inference (s)	GPU memory (GB)
PeMS03	28.1	4.8	1.2
PeMS04	16.6	3.1	1.1
PeMS07	91.9	27.0	1.6
PeMS08	14.2	2.2	1.1

## Conclusions

In this paper, we propose a novel spatial–temporal combination and multi-head flow-attention network (STCMFA) for traffic flow prediction. Extensive experiments were conducted on four real traffic datasets, and the experiment results were compared and analyzed with baseline models, and the following conclusions were obtained:

1. The traditional attention mechanism often suffers from attention degradation when dealing with long sequence data. Although the introduction of specific inductive biases can avoid this problem, it abandons the universality and expressiveness of the model. Therefore, this paper introduces the flow attention mechanism based on the “flow conservation” theory, which avoids the influence of specific induction biases. Its special source competition mechanism and sink allocation mechanism replace the traditional attention weight calculation module, which effectively solves the quadratic complexity problem faced by traditional attention. The experiment results show that flow attention has better prediction performance.
2. There are usually complex nonlinear relationships in traffic flow data, and the traditional linear transformation method in flow attention cannot effectively capture them. Therefore, we design a temporal sequence multi-head flow attention (TS-MFA) module, which uses the temporal model GRU to replace the traditional fully connected layer for nonlinear feature mapping, thereby enhancing the ability of flow attention to model temporal correlation and effectively capturing long-term dependencies in the sequence. The experiment results show that the proposed model has a significant effect in long step prediction.
3. The change of traffic flow data will be affected by time and space at the same time. Therefore, this paper combines the TS-MFA module with GCN to effectively capture the hidden spatial–temporal correlation in traffic flow. In addition, the residual mechanism and feature aggregation strategy are introduced to promote the transmission of feature information and further improve the performance of the model.
4. Through ablation experiments, we prove that each key module in STCMFA plays an active role in traffic flow prediction tasks. The experiment results show that the prediction effect of STCMFA on the four datasets is significantly better than that of the baseline models, which fully proves that the model has strong generalization ability and prediction performance.

Although the proposed model has achieved some research results, it still has some shortcomings. For example, there are different traffic patterns between roads in the traffic network, and the shared parameters in the model cannot model these differences well. In the future, we plan to capture the specific traffic patterns of each road by assigning independent parameters to different roads. In addition, we can try to integrate additional environmental data such as weather and climate into the model as feature information, and further improve the expression ability and prediction accuracy of the model from the perspective of data-driven.

## Data Availability

Data are available from the corresponding author upon request.

## Code availability

The code and visualization program has been published on GitHub (10.5281/zenodo.10902016). There are no access restrictions.

## References

[1] Tyagi AK, Sreenath N, Tyagi AK, Sreenath N (2022). Introduction to intelligent transportation system. Intelligent Transportation Systems: Theory and Practice.

[2] Owais M (2022). Traffic sensor location problem: Three decades of research. Expert Syst. Appl..

[3] Jiang W, Luo J (2022). Graph neural network for traffic forecasting: A survey. Expert Syst. Appl..

[4] Cao S, Wu L, Wu J, Wu D, Li Q (2022). A spatio-temporal sequence-to-sequence network for traffic flow prediction. Inf. Sci..

[5] Su Z, Liu T, Hao X, Hu X (2023). Spatial–temporal graph convolutional networks for traffic flow prediction considering multiple traffic parameters. J. Supercomput..

[6] Fu X (2022). Spatial heterogeneity and migration characteristics of traffic congestion—A quantitative identification method based on taxi trajectory data. Phys. A Stat. Mech. Appl..

[7] Hochreiter S, Schmidhuber J (1997). Long short-term memory. Neural Comput..

[8] Song C, Lin Y, Guo S, Wan H (2020). Spatial-temporal synchronous graph convolutional networks: A new framework for spatial–temporal network data forecasting. Proc. AAAI Conf. Artif. Intell..

[9] Fang, Z., Long, Q., Song, G. & Xie, K. Spatial-temporal graph ODE networks for traffic flow forecasting. In *Proc. 27th ACM SIGKDD Conference on Knowledge Discovery & Data Mining* 364–373. 10.1145/3447548.3467430 (2021).

[10] Zhang R (2022). Spatial–temporal dynamic semantic graph neural network. Neural Comput. Appl..

[11] Sun X, Chen F, Wang Y, Lin X, Ma W (2023). Short-term traffic flow prediction model based on a shared weight gate recurrent unit neural network. Phys. A Stat. Mech. Appl..

[12] Zhou T, Huang B, Li R, Liu X, Huang Z (2022). An attention-based deep learning model for citywide traffic flow forecasting. Int. J. Dig. Earth.

[13] Wang Y, Jing C, Xu S, Guo T (2022). Attention based spatiotemporal graph attention networks for traffic flow forecasting. Inf. Sci..

[14] Lin J, Lin C, Ye Q (2023). Attention based convolutional networks for traffic flow prediction. Multimedia Tools Appl..

[15] Kacham, P., Mirrokni, V. S. & Zhong, P. J. A. PolySketchFormer: Fast transformers via sketches for polynomial kernels. http://arXiv.org/abs/2310.01655 (2023).

[16] Liu J, Guan W (2004). A summary of traffic flow forecasting methods. J. Highw. Transp. Res. Dev..

[17] Yao R, Zhang W, Zhang L (2020). Hybrid methods for short-term traffic flow prediction based on ARIMA-GARCH model and wavelet neural network. J. Transp. Eng. A Syst..

[18] Lint HV, Hinsbergen CPIJ (2012). Short-term traffic and travel time prediction models. Artif. Intell. Appl. Crit. Transp. Issues.

[19] Jeong YS, Byon YJ, Castro-Neto MM, Easa SM (2013). Supervised weighting-online learning algorithm for short-term traffic flow prediction. IEEE Trans. Intell. Transp. Syst..

[20] Cho, K. *et al. Learning Phrase Representations Using RNN Encoder–Decoder for Statistical Machine Translation*. 10.3115/v1/D14-1179 (2014).

[21] Shi, X. *et al.* Convolutional LSTM network: A machine learning approach for precipitation nowcasting. In *Proc. 28th International Conference on Neural Information Processing Systems*, Vol. 1, 802–810 (2015).

[22] Liu, Y., Zheng, H., Feng, X. & Chen, Z. Short-term traffic flow prediction with Conv-LSTM. In *2017 9th International Conference on Wireless Communications and Signal Processing (WCSP)* 1–6. 10.1109/WCSP.2017.8171119 (2017).

[23] Zhang, J., Zheng, Y. & Qi, D. Deep spatio-temporal residual networks for citywide crowd flows prediction. In *AAAI Conference on Artificial Intelligence* (2016).

[24] Yao, H. *et al.* Deep multi-view spatial-temporal network for taxi demand prediction. In *Proc. Thirty-Second AAAI Conference on Artificial Intelligence and Thirtieth Innovative Applications of Artificial Intelligence Conference and Eighth AAAI Symposium on Educational Advances in Artificial Intelligence* 316 (2018).

[25] Xu C, Zhang A, Xu C, Chen Y (2022). Traffic speed prediction: Spatio-temporal convolution network based on long-term, short-term and spatial features. Appl. Intell..

[26] Niepert, M., Ahmed, M. & Kutzkov, K. Learning convolutional neural networks for graphs. In *Proc. 33rd International Conference on International Conference on Machine Learning*, Vol. 48, 2014–2023 (2016).

[27] Li, Y., Yu, R., Shahabi, C. & Liu, Y. J. A. L. *Diffusion Convolutional Recurrent Neural Network: Data-Driven Traffic Forecasting* (2017).

[28] Yu, B., Yin, H. & Zhu, Z. *Spatio-Temporal Graph Convolutional Networks: A Deep Learning Framework for Traffic Forecasting* (2018).

[29] Wu, Z., Pan, S., Long, G., Jiang, J. & Zhang, C. *Graph WaveNet for Deep Spatial–Temporal Graph Modeling* (2019).

[30] Bai, L., Yao, L., Li, C., Wang, X. & Wang, C. Adaptive graph convolutional recurrent network for traffic forecasting. In *Proc. 34th International Conference on Neural Information Processing Systems* 1494 (2020).

[31] Lan, S. *et al. DSTAGNN: Dynamic Spatial-Temporal Aware Graph Neural Network for Traffic Flow Forecasting* (2022). 10.1016/j.neunet.2026.108989 42001625

[32] Tan Z, Zhu Y, Liu B (2023). Learning spatial–temporal feature with graph product. Signal Process..

[33] Xue J, Zheng T, Han J (2021). Exploring attention mechanisms based on summary information for end-to-end automatic speech recognition. Neurocomputing.

[34] Kong X, Zhang J, Wei X, Xing W, Lu W (2022). Adaptive spatial-temporal graph attention networks for traffic flow forecasting. Appl. Intell..

[35] Xu, K. *et al.* Show, attend and tell: Neural image caption generation with visual attention. In *Proc. 32nd International Conference on International Conference on Machine Learning*, Vol. 37, 2048–2057 (2015).

[36] Velickovic, P. *et al.* Graph attention networks. http://arXiv.org/abs/1710.10903 (2017).

[37] Guo, S., Lin, Y., Feng, N., Song, C. & Wan, H. Attention based spatial-temporal graph convolutional networks for traffic flow forecasting. In *Proc. Thirty-Third AAAI Conference on Artificial Intelligence and Thirty-First Innovative Applications of Artificial Intelligence Conference and Ninth AAAI Symposium on Educational Advances in Artificial Intelligence* 114. 10.1609/aaai.v33i01.3301922 (2019).

[38] Li, H. *et al.* DetectorNet: Transformer-enhanced spatial temporal graph neural network for traffic prediction. In *Proc. 29th International Conference on Advances in Geographic Information Systems* 133–136. 10.1145/3474717.3483920 (2021).

[39] Zhang, M., Zhou, W., Huang, J., Huang, K. & Tang, X. Self-attention based chebnet recurrent network for traffic forecasting. In *Proc. 2022 Chinese Intelligent Systems Conference* 300–309 (2022).

[40] Qin, Z. *et al.* cosFormer: Rethinking softmax in attention. http://arXiv.org/abs/2202.08791 (2022).

[41] Wu, H., Wu, J., Xu, J., Wang, J. & Long, M. Flowformer: Linearizing transformers with conservation flows. In *International Conference on Machine Learning* (2022).

[42] Bruna, J., Zaremba, W., Szlam, A. & Lecun, Y. *Spectral Networks and Locally Connected Networks on Graphs* (2013).

[43] Bai, L., Yao, L., Kanhere, S. S., Wang, X. & Sheng, Q. Z. STG2seq: Spatial-temporal graph to sequence model for multi-step passenger demand forecasting. In *Proc. 28th International Joint Conference on Artificial Intelligence* 1981–1987 (2019).

[44] Lai Q, Chen P (2024). LEISN: A long explicit–implicit spatio-temporal network for traffic flow forecasting. Expert Syst. Appl..

